# Quantitative Analysis of Residual COVID-19 Lung CT Features: Consistency among Two Commercial Software

**DOI:** 10.3390/jpm11111103

**Published:** 2021-10-28

**Authors:** Vincenza Granata, Stefania Ianniello, Roberta Fusco, Fabrizio Urraro, Davide Pupo, Simona Magliocchetti, Fabrizio Albarello, Paolo Campioni, Massimo Cristofaro, Federica Di Stefano, Nicoletta Fusco, Ada Petrone, Vincenzo Schininà, Alberta Villanacci, Francesca Grassi, Roberta Grassi, Roberto Grassi

**Affiliations:** 1 Division of Radiology, Istituto Nazionale Tumori IRCCS Fondazione Pascale-IRCCS di Napoli, 80131 Naples, Italy; v.granata@istitutotumori.na.it; 2 Radiology Unit, National Institute for Infectious Diseases Lazzaro Spallanzani IRCCS, 00149 Rome, Italy; stefianni66@gmail.com (S.I.); Fabrizio.albarello@inmi.it (F.A.); paolo.campioni@inmi.it (P.C.); massimo.cristofaro@inmi.it (M.C.); federica.distefano@inmi.it (F.D.S.); nicoletta.fusco@inmi.it (N.F.); ada.petrone@inmi.it (A.P.); vincenzo.schinina@inmi.it (V.S.); alberta.villanacci@inmi.it (A.V.); 3 Medical Oncology Division, Igea SpA, 80013 Naples, Italy; 4 Division of Radiology, Università degli Studi della Campania Luigi Vanvitelli, 80125 Naples, Italy; fabrizio.urraro@unicampania.it (F.U.); dave.dp93@gmail.com (D.P.); simonamag7@gmail.com (S.M.); francescagrassi1996@gmail.com (F.G.); grassi.roberta89@gmail.com (R.G.); roberto.grassi@unicampania.it (R.G.); 5 Italian Society of Medical and Interventional Radiology (SIRM), SIRM Foundation, Via della Signora 2, 20122 Milan, Italy

**Keywords:** COVID-19, post COVID-19 sequelae, computed tomography, quantitative analysis, artificial intelligence

## Abstract

Objective: To investigate two commercial software and their efficacy in the assessment of chest CT sequelae in patients affected by COVID-19 pneumonia, comparing the consistency of tools. Materials and Methods: Included in the study group were 120 COVID-19 patients (56 women and 104 men; 61 years of median age; range: 21–93 years) who underwent chest CT examinations at discharge between 5 March 2020 and 15 March 2021 and again at a follow-up time (3 months; range 30–237 days). A qualitative assessment by expert radiologists in the infectious disease field (experience of at least 5 years) was performed, and a quantitative evaluation using thoracic VCAR software (GE Healthcare, Chicago, Illinois, United States) and a pneumonia module of ANKE ASG-340 CT workstation (HTS Med & Anke, Naples, Italy) was performed. The qualitative evaluation included the presence of ground glass opacities (GGOs) consolidation, interlobular septal thickening, fibrotic-like changes (reticular pattern and/or honeycombing), bronchiectasis, air bronchogram, bronchial wall thickening, pulmonary nodules surrounded by GGOs, pleural and pericardial effusion, lymphadenopathy, and emphysema. A quantitative evaluation included the measurements of GGOs, consolidations, emphysema, residual healthy parenchyma, and total lung volumes for the right and left lung. A chi-square test and non-parametric test were utilized to verify the differences between groups. Correlation coefficients were used to analyze the correlation and variability among quantitative measurements by different computer tools. A receiver operating characteristic (ROC) analysis was performed. Results: The correlation coefficients showed great variability among the quantitative measurements by different tools when calculated on baseline CT scans and considering all patients. Instead, a good correlation (≥0.6) was obtained for the quantitative GGO, as well as the consolidation volumes obtained by two tools when calculated on baseline CT scans, considering the control group. An excellent correlation (≥0.75) was obtained for the quantitative residual healthy lung parenchyma volume, GGO, consolidation volumes obtained by two tools when calculated on follow-up CT scans, and for residual healthy lung parenchyma and GGO quantification when the percentage change of these volumes were calculated between a baseline and follow-up scan. The highest value of accuracy to identify patients with RT-PCR positive compared to the control group was obtained by a GGO total volume quantification by thoracic VCAR (accuracy = 0.75). Conclusions: Computer aided quantification could be an easy and feasible way to assess chest CT sequelae due to COVID-19 pneumonia; however, a great variability among measurements provided by different tools should be considered.

## 1. Introduction

A new coronavirus (severe acute respiratory syndrome coronavirus 2, SARS-CoV-2) is the pathogen responsible for the SARS-CoV-2 disease (COVID-19), which has spread throughout the world since December 2019 [1,2,3,4,5,6,7,8,9]. COVID-19 was defined as a pandemic by the World Health Organization on 11 March 2020 [10]. The clinical expressions of COVID-19 range from flu-like symptoms to respiratory failure, the management of which demands advanced respiratory assistance and artificial ventilation [11,12,13,14,15,16,17,18,19,20,21]. The clinical spectrum of COVID-19 pneumonia ranges from mild to critical cases, among which the diagnosis of ordinary, severe, and critical cases was related to chest computed tomography (CT) findings [22,23]. CT imaging allows for the early detection of lung abnormalities in patients with SARS-CoV-2 pneumonia [24,25], representing a useful diagnostic tool, with pooled sensitivity and a specificity of 94% and 37%, respectively [26]. Additionally, approximately one-third of COVID-19 survivors showed pulmonary fibrotic-like changes at a six-month follow-up chest CT [27]; there is speculation that some of these findings will resolve over time, and are therefore not fibrosis [27]. Although a visual method allows the assessment of these findings, a quantitative evaluation based on software systems, not dependent on the experience of the reader, allows for a greater accuracy of analysis and facilitates the evaluation of the data over time, reducing the error of the qualitative evaluation alone [8]. While several artificial intelligence (AI) models have been developed to facilitate the automation of COVID-19 diagnosis [11,13,17], there has been little study of COVID-19 lesion segmentation. To detect regions of interest (ROIs) from CT scans is an interesting and challenging task for several reasons: (a) a large divergence in the characteristics of lesions in terms of scope, location, shape, and quality makes them difficult to classify; (b) small, inter-class divergence means that the margins of ground-glass opacity (GGO) predominantly exhibit clouded manifestation and low contrast, which complicates the detection process; (c) noisy annotation is inevitable for rare or new diseases (e.g., COVID-19), which decreases segmentation efficiency. However, the quantitative assessment of infection and longitudinal changes in CT findings could offer useful and vital information in fighting against COVID-19.

The aim of this retrospective study is to investigate the efficacy of two commercial software in the assessment of chest CT sequelae in patients affected by COVID-19 pneumonia, comparing the consistency of these two tools.

## 2. Materials and Methods

### 2.1. Patient Selection

This retrospective study included patients enrolled by the National Institute of Infectious Diseases Lazzaro Spallanzani Hospital, Rome, Italy.

Considering the emergency period, the local institutional review board waived informed consent for included patients in this retrospective study.

In order to homogenize the sample under examination, only patients who were subjected to CT at discharge and at a 3-month follow-up (range 30–237 days) were included.

The study group included 120 patients (56 women and 104 men; median age: 61 years; range: 21–93 years) who were confirmed to be infected with COVID-19 using the nucleic acid amplification test in the respiratory tract with a reverse transcription real-time fluorescence polymerase chain reaction test (RT-PCR) between 5 March 2020 and 15 March 2021.

As a control group, we selected 40 patients (median age: 60 years; range: 38–90) without lung disease who underwent chest CT at the same institute that was staging an examination for colorectal cancer.

### 2.2. CT Technique

We performed 128 slices of chest CT scans with Incisive Philips CT scanners (Amsterdam, The Netherlands). CT examinations were performed with the patient in the supine position in breath-hold, and inspiration using a standard dose protocol, without contrast intravenous injection. The scanning range was from the apex to the base of the lungs. The tube voltage and the current tube were 120 kV and 100–200 mA (and if applicable, using *z*-axis tube current modulation), respectively. All data were reconstructed with a 0.6–1.0 mm increment. The matrix was 512 mm × 512 mm. Images were reconstructed using a sharp reconstruction kernel for parenchyma evaluation and hard reconstruction kernel for other lung evaluation. All data were reconstructed with a 0.6–1.0 mm increment. Multiplanar reconstruction was also calculated. Details are provided in previous papers [8,11].

### 2.3. Qualitative Assessment

Four expert radiologists in the infectious disease field (with experience of at least 5 years) were working independently on the same CT series of studies, and in addition, discrepant findings were recorded and evaluated in consensus. A qualitative evaluation included the presence of the following CT findings: (a) GGOs, (b) consolidation, (c) interlobular septal thickening, (d) fibrotic-like changes (reticular pattern and/or honeycombing), (e) bronchiectasis, (d) air bronchogram, (e) bronchial wall thickening, (f) pulmonary nodules surrounded by GGOs, (g) pleural and (h) pericardial effusion, (i) lymphadenopathy (defined as lymph node with short axis > 10 mm), and (j) emphysema.

All chest CT findings were defined according to the Fleischner Society glossary [28].

For each of them, they reported (1) location, (2) multilobe involvement, (3) total lobar involvement, and (4) bilateral distribution.

### 2.4. CT Post-Processing

Primary image data sets (0.6–1.0 mm) were transferred to the PACS workstation and the same CT images were evaluated using two clinically available computer tools by the same 4 readers in consensus (no discrepant data can be obtained with automatic computerized quantification). The tools used were thoracic VCAR software (GE Healthcare, Chicago, IL, USA) and a pneumonia module of ANKE ASG-340 CT workstation (HTS Med & Anke, Naples, Italy). Table 1 reports a comparison among evaluated commercial software based on the provided functionalities.

#### 2.4.1. Post-Processing with Thoracic VCAR Software

**Figure 1:**
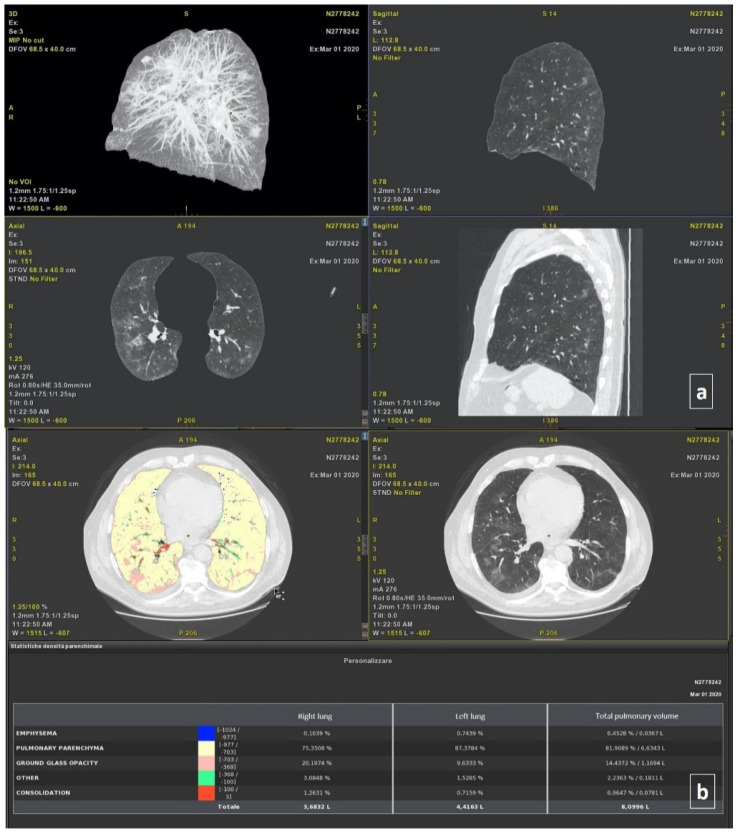
Automatic segmentation of thoracic disease by COVID-19 using the Thoracic VCAR Tool of General Electric Healthcare: (**a**) 3D axial and sagittal plane reconstruction; (**b**) density analysis of parenchyma. This case had bilateral and diffuse, and consolidations in multiple lobes.

Thoracic VCAR software is a CE-marked medical device designed to quantify pulmonary emphysema in patients with chronic obstructive pulmonary disease. The tool provides segmentation of the lungs and of the airway tree. Moreover, the tools provided the quantification of the emphysema, healthy residual lung parenchyma, GGO, and consolidation based on a Hounsfield unit. Details are provided in previous papers [8,11]. The total volumes for both the right and left lung were also calculated (Figure 1).

#### 2.4.2. Post-Processing with ANKE ASG-340 CT Workstation

**Figure 2:**
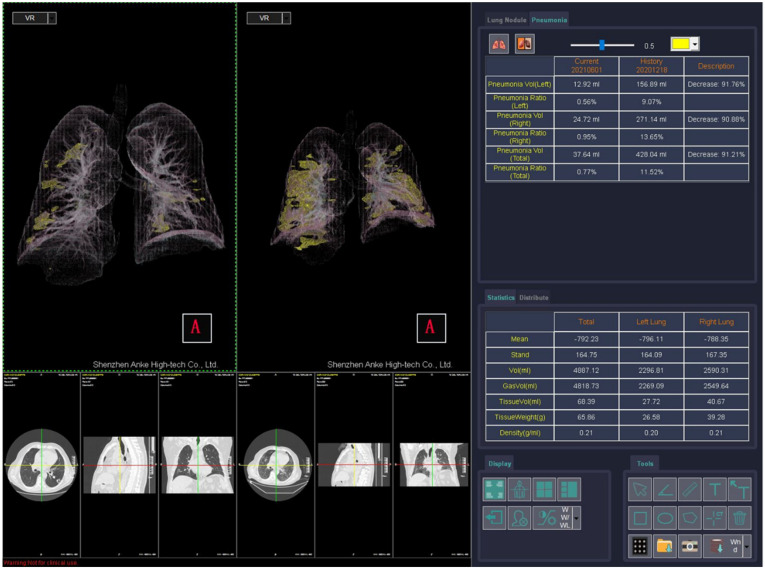
Automatic Segmentation of Thoracic Disease by COVID-19 using the pneumonia tool of ANKE ASG-340 CT workstation.

The ANKE ASG-340 CT workstation from HTS Med & ANKE is a comprehensive CT workstation that uses lung nodules analysis, pneumonia analysis, dental pack, vascular analysis cerebral hemorrhage analysis, and so on. The pneumonia module is designed to quantify pneumonia patients. The software provides automatic segmentation of the lungs and lung lobs and automatic location and measurement pneumonia including volume, CT value, and component analysis. It provides the classification of voxels based on Hounsfield Units (Figure 2), as was previously described for the thoracic VCAR Tool.

### 2.5. Statistical Analysis

The median value and range were calculated. A chi-square test, Mann–Whitney test, and Kruskal–Wallis test were used to verify the differences between groups. The Pearson correlation coefficient and intraclass correlation coefficient were used to analyze the correlation and variability among the quantitative measurements generated by different tools [3].

A receiver operating characteristic (ROC) analysis was performed. The area under curve (AUC), sensitivity, specificity, positive predictive value, negative predictive value, and accuracy were obtained. A *p* value of <0.05 was considered significant for all tests.

The statistical analyses were performed using the Statistics Toolbox of MATLAB R2007a (MathWorks, Natick, MA, USA).

## 3. Results

In the study group, 240 chest CT examinations (at discharge/baseline and follow-up time; range: 30–237 days) were analyzed.

### 3.1. Qualitative Assessment

At baseline, the patients had: GGOs (120; 100%); consolidation (108; 90.0%); interlobular septal thickening (120; 100%); fibrotic-like changes (reticular pattern and/or honeycombing) (116; 96.7%); bronchiectasis (80; 66.7%); air bronchogram (10; 8.3%); bronchial wall thickening (120; 100%); pulmonary nodules surrounded by GGOs (40; 33.3%); pleural (45; 37.5%) and pericardial effusion (6; 5%); lymphadenopathy (0; 0%), and emphysema (107; 89.2%).

All patients had a multilobe and bilateral distribution.

At follow-up, the patients had: GGOs (120; 100%); consolidation (120; 100%); interlobular septal thickening (120; 100%); fibrotic-like changes (reticular pattern and/or honeycombing) (120; 100%); bronchiectasis (120; 100%); air bronchogram (40; 33.3%); bronchial wall thickening (120; 100%); pulmonary nodules surrounded by GGOs (0; 0%); pleural (4; 3.3%) and pericardial effusion (0; 0%), and emphysema (107; 89.2%).

A statistically significant difference was found considering the presence in the percentage value of pulmonary nodules surrounded by GGOs pleural effusion between the two groups (*p* < 0.01 at Chi square test).

All patients had a bilateral distribution with multilobe involvement.

In the control group, we evaluated 40 chest CT examinations in 12 patients (30%), and the only features identified was emphysema.

### 3.2. Quantitative Assessment

The thoracic VCAR software was not able to perform volume segmentation in 12/280 (4.3%) cases, while the pneumonia module of the ANKE ASG-340 CT workstation performed in 19/280 (6.8%) patients.

The ICC showed great variability among the quantitative measurements of the emphysema, residual healthy lung parenchyma volume, GGO, and consolidations volumes obtained by different tools when calculated on baseline CT scans (Table 2), and considering all patients.

A good ICC (≥0.6) was obtained for the quantitative GGO and consolidations volumes obtained by two tools when calculated on baseline CT scans (Table 2), and considering the control group (Table 2).

An excellent ICC (≥0.75) was obtained for the quantitative residual healthy lung parenchyma, GGO, and consolidations volumes obtained by two tools when calculated on follow-up CT scans (Table 3).

In addition, an excellent ICC (≥0.75) was obtained for the residual healthy lung parenchyma volume and GGO quantifications when the percentage change of these volumes was calculated between the baseline and follow-up examination.

The lowest variability in the quantification was obtained for the GGO volume quantification (ICC = 0.94). The Pearson correlation analyses (Table 4) showed a low correlation for each of the quantitative volume measurements determined by the thoracic VCAR tool and ANKE ASG-340 CT workstation pneumonia tool; exclusively, the GGO measurement showed a moderate correlation (Pearson correlation coefficient = 0.682, *p* < 0.01).

**Figure 3:**
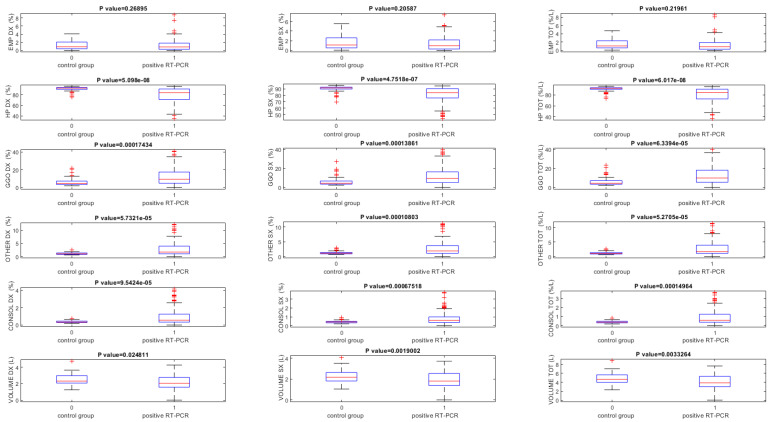
Boxplot of lung volumes quantified using the thoracic VCAR obtained on baseline CT scans. Note. EMP = emphysema; HP = health parenchyma; GGO = ground-glass opacity; CONSOL = consolidations.

The lung volumes quantified using the thoracic VCAR tool on baseline CT scans were significantly different between RT-PCR positive and the control group (*p* < 0.05) for all volumes, except that for the quantification of the emphysema volume (Table 5, Figure 3).

**Figure 4:**
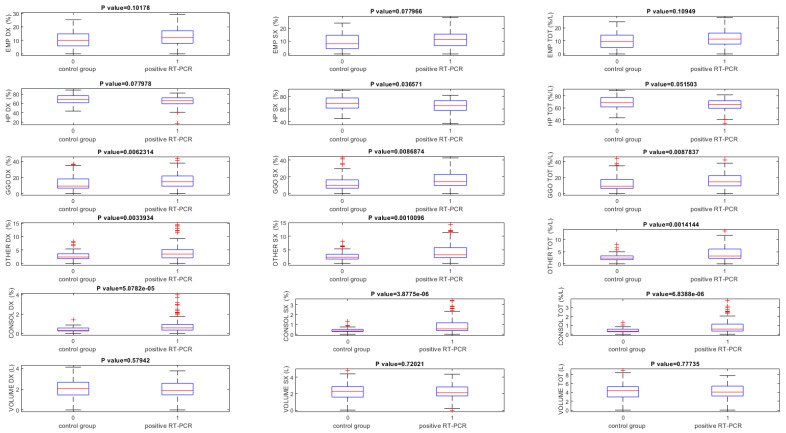
Boxplot of lung volumes quantified using the ANKE ASG-340 CT workstation pneumonia tool obtained on baseline CT scans. Note. EMP = emphysema; HP = health parenchyma; GGO = ground-glass opacity; CONSOL = consolidations.

Instead, using ANKE ASG-340 CT pneumonia software baseline CT scans, GGO and consolidation volumes exclusively showed statistically significant differences among patients with RT-PCR positive and the control group (*p* < 0.05) (Table 6, Figure 4).

Table 7 shows the volumes percentage change between baseline and follow-up time in patients with positive RT-PCR in terms of median, minimum, and maximum values.

The lung volumes quantified by two tools in terms of median, minimum, and maximum values obtained on follow-up CT scans are reported in the Table 8.

Table 9 showed ROC analysis results for volumes obtained on baseline CT scans for both tools. The highest value of accuracy to identify patients with RT-PCR positive was obtained by GGO total volume quantification by the thoracic VCAR (accuracy = 0.75).

Considering the results obtained by the ANKE ASG-340 CT pneumonia tool, the consolidation volume of the left lung obtained the highest accuracy, equal to 0.

## 4. Discussions and Conclusions

In this study, we evaluated the quantitative analysis efficacy of chest CT sequelae in patients affected by COVID-19 pneumonia, comparing the consistency of two computerized tools. The visual evaluation of longitudinal changes in CT scans by radiologists is often a tedious task. There is a need to have a simple and fast automated method that can provide the segmentation and quantification of infection regions in order to evaluate the progression of the infected patients using lung CT scans [29,30,31,32,33,34,35]. Additionally, an objective evaluation by means of AI systems allows a data quantification, and therefore, an accurate definition of the disease progression; this is an element that otherwise is not very robust if entrusted to a simple visual inspection [36,37,38]. Recently, several computer tools have been proposed for the recognition of lung lesions from COVID-19 on CT examination [39,40,41]. However, many of them are not approved as medical devices, nor do they have the CE marking. Furthermore, the variability reported in the results obtained by these tools makes it difficult to choose the most accurate system [8].

To the best of our knowledge, this manuscript is the first with the aim to compare different computer tools for chest CT sequelae in patients affected by COVID-19 pneumonia. We demonstrated that there was a great variability among the quantitative measurements of the emphysema, residual healthy lung parenchyma volume, GGO, and consolidations volumes obtained by different computer tools when calculated on baseline CT scans. Instead, a good ICC was obtained for the quantitative measurements of the GGO and consolidations volumes obtained by two different computer tools when calculated on baseline CT scans, while considering the control group. Moreover, an excellent ICC was obtained for the quantitative measurements of the residual healthy lung parenchyma volume, GGO, and consolidations volumes obtained by two different computer tools when calculated on follow-up CT scans, and for the residual healthy lung parenchyma volume and GGO quantifications when the percentage change of these volumes was calculated between the baseline and follow-up scan. The lowest variability in the quantification was obtained for the GGO volume.

The Pearson correlation analyses showed a low correlation between the quantitative volume measurements determined by the thoracic VCAR tool and ANKE ASG-340 CT workstation pneumonia tool; exclusively, the measurement of the GGO showed a moderate correlation.

We think that the greater variability found at the baseline is linked to the complexity of the cases analyzed in this phase, which could affect the accuracy of lesion segmentation. As demonstrated by Herrmann et al. [42], in ARDS, image segmentation is especially difficult, since in some cases, it is almost impossible to discriminate the edge of the lung parenchyma from a pleural effusion, particularly in the most dependent lung regions and most severe ARDS forms. Also, at different airway pressures, they observed differences in lung weights. These variations may result either from the segmentation procedure and/or from actual changes in lung weight, primarily due to a possible airway pressure-dependent blood shift. It is unfortunately impossible to determine how much of the weight variation is due to an intrathoracic blood shift or to inaccuracies of the segmentation process. The decrease in intrathoracic blood volume we estimated in a previous work with increasing airway pressures was about 100 mL, leading to a small decrease in lung weight [43].

So, we believe that at follow-up, with a smaller extension of pulmonary involvement, the variability between the two systems is partially reduced, since the segmentation process is simpler in the absence of variables related to the presence of pleural effusion, and increase in pressures in the pulmonary vessels; the resolution of these variables favor the definition of the different pixels [44].

There were main critical points of the thoracic VCAR tool: automatic segmentation does not include areas of abundant consolidations of the lung parenchyma or pleural effusions, if conspicuous, requiring the manual segmentation modality; there was difficulty in the manual lung segmentation mode; its correction, performed on a single slice, takes time.

There were main critical points of the ANKE ASG-340 CT workstation pneumonia tool: it is slow in the analysis (120 s of median value compared to 10 s); it overestimates emphysema quantification; it is not able to segment complex cases with conspicuous effusion and/or areas of extensive consolidations.

Both tools, moreover, do not recognize several CT findings typical of the evolution of the disease, such as: (a) interlobular septal thickening, (b) fibrotic-like changes (reticular pattern and/or honeycombing), (c) bronchiectasis, (d) air bronchogram, (e) bronchial wall thickening, (f) pulmonary nodules surrounded by GGOs, (g) pleural and (h) pericardial effusion, and (i) lymphadenopathy, including these feature in others and reducing the accuracy of the assessment of the fibrotic-like changes.

According to Johns Hopkins University, case-fatality rates of COVID-19 patients ranges between 1% and 7% based on days since first confirmed case, testing efficacy, local pandemic response policies, and the population age [45,46,47,48,49]. Multi-organ manifestations of COVID-19 are now well-documented [50,51,52,53,54,55,56,57], but the potential long-term implications of these manifestations remain to be uncovered. Several studies have reported impaired exercise capacity and diffusing capacity for carbon monoxide (DL_CO_) in SARS-CoV-1 survivors extending from 6 months to 15 years of follow-up [58,59,60,61,62,63,64], suggesting impairment of the intra-alveolar diffusion pathway. In this scenario, it is clear that it is important to have tools that objectively allow a stratification of patients based on the risk of developing chronic diseases that can impact their quality of life, and economically impact health care [65,66]. We believe that the computed assessment of CT findings could identify pulmonary abnormalities and lung recruitment, and we believe that knowledge of the percentage of potentially recruitable lung evolution may be important to establish the therapeutic management in chest sequelae in patients affected by COVID-19 pneumonia.

The present study has advantages: first, the homogeneity of the sample under examination and the follow-up at three months; second, the CT was performed at the same center, reducing the variability linked to different equipment; third, the high level of expertise of the group of radiologists who analyzed the images.

The major technical limitations for both tools is the lack of correlation of radiological data with clinical/functional data. It would be useful to evaluate how CT findings relate to functional investigations such as spirometry and/or lung scintigraphy. However, these data are present only for a small part of the population under examination.

In summary, computer-aided quantification could be an easy and feasible way to assess chest CT sequelae due to COVID-19 pneumonia; however, a great variability among the measurements provided by different tools should be considered.

## Figures and Tables

**Table 1:** Description of computed based tool functionalities.

Functionalities	Thoracic VCAR	ANKE ASG-340 CT Workstation
Quantitative data for each lobe	no	yes
Manually segmentation	yes	no
Preliminary possibility to exclude airways	yes	no
CE marking for lung study	yes	no
Evaluation separately pleural effusion	no	no
Unstructured report	yes	yes
Combined structured report	no	yes
Proportion of pneumonia lesion measurement	no	yes
Comparison among exams	no	yes

**Table 2:** The intraclass coefficient (ICC) among quantitative volumes obtained using different commercial computerized tools on baseline CT scans.

Variability		EMP DX (%)	EMP SX (%)	EMP TOT (%/L)	HP DX (%)	HP SX (%)	HP TOT (%/L)	GGO DX (%)	GGO SX (%)	GGO TOT (%/L)	OTHER DX (%)	OTHER SX (%)	OTHER TOT (%/L)	CONSOL DX (%)	CONSOL SX (%)	CONSOL TOT (%/L)	VOLUME DX (L)	VOLUME SX (L)	VOLUME TOT (L)
**All patients**	ICC	0.04	0.04	0.04	0.11	0.08	0.08	0.07	0.05	0.07	0.05	0.05	0.05	0.05	0.05	0.05	0.00	0.00	0.00
	Lower Bound	−0.04	−0.04	−0.04	−0.03	−0.04	−0.04	−0.07	−0.09	−0.07	−0.10	−0.09	−0.09	−0.11	−0.10	−0.10	−0.02	−0.02	−0.02
	Upper Bound	0.14	0.14	0.15	0.25	0.21	0.21	0.22	0.19	0.21	0.19	0.20	0.19	0.20	0.21	0.21	0.03	0.03	0.03
**RT-PCR positive**	ICC	0.04	0.02	0.03	0.06	0.04	0.04	0.03	0.01	0.02	0.00	0.00	−0.01	0.00	0.00	0.00	0.00	0.00	0.00
	Lower Bound	−0.05	−0.05	−0.05	−0.08	−0.10	−0.10	−0.15	−0.17	−0.15	−0.17	−0.17	−0.18	−0.18	−0.18	−0.18	−0.03	−0.03	−0.02
	Upper Bound	0.14	0.11	0.13	0.21	0.18	0.18	0.20	0.18	0.19	0.17	0.17	0.16	0.18	0.18	0.18	0.03	0.03	0.03
**Control group**	ICC	0.06	0.09	0.08	0.06	0.04	0.05	**0.60**	**0.68**	**0.60**	0.51	0.10	0.11	0.68	0.66	0.68	0.00	0.00	0.00
	Lower Bound	−0.06	−0.07	−0.07	−0.05	−0.06	−0.06	**−0.07**	**−0.08**	**−0.07**	−0.08	−0.09	−0.08	0.09	0.06	0.09	−0.03	−0.02	−0.03
	Upper Bound	0.23	0.31	0.27	0.23	0.18	0.20	**0.77**	**0.74**	**0.76**	0.53	0.32	0.33	0.81	0.80	0.81	0.05	0.04	0.04

Note. EMP = emphysema; HP = health parenchyma; GGO = ground-glass opacity; CONSOL = consolidations; ICC = intraclass coefficient.

**Table 3:** The intraclass coefficient (ICC) among quantitative volumes obtained using different commercial computerized tools on follow-up CT scans.

Variability	EMP DX (%)	EMP SX (%)	EMP TOT (%/L)	HP DX (%)	HP SX (%)	HP TOT (%/L)	GGO DX (%)	GGO SX (%)	GGO TOT (%/L)	OTHER DX (%)	OTHER SX (%)	OTHER TOT (%/L)	CONSOL DX (%)	CONSOL SX (%)	CONSOL TOT (%/L)	VOLUME DX (L)	VOLUME SX (L)	VOLUME TOT (L)
**In follow-up CT scans**																		
**ICC**	0.18	0.20	0.19	**0.87**	**0.87**	**0.85**	**0.82**	**0.83**	**0.94**	0.63	0.71	0.68	0.02	**0.90**	**0.91**	0.00	0.00	0.00
**Lower Bound**	−0.06	−0.06	−0.06	**0.46**	**0.60**	**0.40**	**0.62**	**0.66**	**0.63**	0.14	0.18	0.14	−0.14	**0.85**	**0.85**	−0.05	−0.05	−0.05
**Upper Bound**	0.39	0.43	0.42	**0.95**	**0.94**	**0.94**	**0.90**	**0.90**	**0.92**	0.82	0.87	0.85	0.17	**0.93**	**0.94**	0.06	0.06	0.06
**Considering percentage change of volume measurements calculated between baseline and follow-up.**																		
**ICC**	0.01	0.02	0.01	**0.75**	**0.75**	**0.78**	**0.75**	**0.75**	**0.76**	0.34	0.30	0.31	0.02	0.06	0.37	0.59	0.35	0.61
**Lower Bound**	−0.15	−0.13	−0.14	**0.63**	**0.61**	**0.02**	**0.21**	**0.16**	**0.19**	0.19	0.15	0.16	−0.15	−0.10	0.23	0.48	0.20	0.49
**Upper Bound**	0.17	0.18	0.17	**0.79**	**0.77**	**0.33**	**0.79**	**0.80**	**0.87**	0.47	0.44	0.45	0.18	0.22	0.50	0.69	0.48	0.70

Note. EMP = emphysema; HP = health parenchyma; GGO = ground-glass opacity; CONSOL = consolidations; ICC = intraclass coefficient.

**Table 4:** Pearson correlation coefficient among quantitative volumes obtained using different tools.

		ThoracicVCAR EMP TOT (%/L)	ThoracicVCAR HP TOT (%/L)	ThoracicVCAR GGO TOT (%/L)	ThoracicVCAR OTHER TOT (%/L)	ThoracicVCAR CONSOL TOT (%/L)	ThoracicVCAR VOLUME TOT (L)	ANKE ASG-340 CT EMP TOT (%/L)	ANKE ASG-340 CT HP TOT (%/L)	ANKE ASG-340 CT GGO TOT (%/L)	ANKE ASG-340 CT OTHER TOT (%/L)	ANKE ASG-340 CT CONSOL TOT (%/L)	ANKE ASG-340 CT VOLUME TOT (L)
ThoracicVCAR EMP TOT (%/L)	Pearson Correlation Coefficient	1	0.056	−0.278 **	−0.208 **	−0.202 **	0.311 **	0.437 **	−0.076	−0.183 **	−0.124 *	−0.067	0.127 *
	*p* value		0.362	0.000	0.001	0.001	0.000	0.000	0.222	0.003	0.048	0.288	0.043
ThoracicVCAR HP TOT (%/L)	Pearson Correlation Coefficient	0.056	1	−0.959 **	−0.895 **	−0.806 **	0.589 **	0.098	0.217 **	−0.336 **	−0.254 **	−0.154 *	0.253 **
	*p* value	0.362		0.000	0.000	0.000	0.000	0.119	0.000	0.000	0.000	0.013	0.000
ThoracicVCAR GGO TOT (%/L)	Pearson Correlation Coefficient	−0.278 **	−0.959 **	1	0.826 **	0.724 **	−0.645 **	−0.208 **	−0.192 **	0.682 **	0.267 **	0.151 *	−0.284 **
	*p* value	0.000	0.000		0.000	0.000	0.000	0.001	0.002	0.000	0.000	0.015	0.000
ThoracicVCAR OTHER TOT (%/L)	Pearson Correlation Coefficient	−0.208 **	−0.895 **	0.826 **	1	0.924 **	−0.526 **	−0.098	−0.164 **	0.250 **	0.236 **	0.163 **	−0.191 **
	*p* value	0.001	0.000	0.000		0.000	0.000	0.117	0.009	0.000	0.000	0.009	0.002
ThoracicVCAR CONSOL TOT (%/L)	Pearson Correlation Coefficient	−0.202 **	−0.806 **	0.724 **	0.924 **	1	−0.485 **	−0.144 *	−0.157 *	0.257 **	0.248 **	0.184 **	−0.197 **
	*p* value	0.001	0.000	0.000	0.000		0.000	0.021	0.012	0.000	0.000	0.003	0.001
ThoracicVCAR VOLUME TOT (L)	Pearson Correlation Coefficient	0.311 **	0.589 **	−0.645 **	−0.526 **	−0.485 **	1	0.197 **	0.122	−0.341 **	−0.231 **	−0.121	0.523 **
	*p* value	0.000	0.000	0.000	0.000	0.000		0.001	0.050	0.000	0.000	0.054	0.000
ANKE ASG-340 CT EMP TOT (%/L)	Pearson Correlation Coefficient	0.437 **	0.098	−0.208 **	−0.098	−0.144 *	0.197 **	1	−0.053	−0.484 **	−0.426 **	−0.335 **	0.372 **
	*p* value	0.000	0.119	0.001	0.117	0.021	0.001		0.391	0.000	0.000	0.000	0.000
ANKE ASG-340 CT HP TOT (%/L)	Pearson Correlation Coefficient	−0.076	0.217 **	−0.192 **	−0.164 **	−0.157 *	0.122	−0.053	1	−0.705 **	−0.701 **	−0.583 **	0.422 **
	*p* value	0.222	0.000	0.002	0.009	0.012	0.050	0.391		0.000	0.000	0.000	0.000
ANKE ASG-340 CT GGO TOT (%/L)	Pearson Correlation Coefficient	−0.183 **	−0.336 **	0.682 **	0.250 **	0.257 **	−0.341 **	−0.484 **	−0.705 **	1	0.839 **	0.625 **	−0.666 **
	*p* value	0.003	0.000	0.000	0.000	0.000	0.000	0.000	0.000		0.000	0.000	0.000
ANKE ASG-340 CT OTHER TOT (%/L)	Pearson Correlation Coefficient	−0.124 *	−0.254 **	0.267 **	0.236 **	0.248 **	−0.231 **	−0.426 **	−0.701 **	0.839 **	1	0.895 * *	−0.572 * *
	*p* value	0.048	0.000	0.000	0.000	0.000	0.000	0.000	0.000	0.000		0.000	0.000
ANKE ASG-340 CT CONSOL TOT (%/L)	Pearson Correlation Coefficient	−0.067	−0.154 *	0.151 *	0.163 **	0.184 **	−0.121	−0.335 **	−0.583 **	0.625 **	0.895 *	1	−0.437 *
	*p* value	0.288	0.013	0.015	0.009	0.003	0.054	0.000	0.000	0.000	0.000		0.000
ANKE ASG-340 CT VOLUME TOT (L)	Pearson Correlation Coefficient	0.127 *	0.253 **	−0.284 **	−0.191 **	−0.197 **	0.523 **	0.372 **	0.422 **	−0.666 **	−0.572 *	−0.437 **	1
	*p* value	0.043	0.000	0.000	0.002	0.001	0.000	0.000	0.000	0.000	0.000	0.000	

Note. EMP = Emphysema; HP = health parenchyma; GGO = ground−glass opacity; CONSOL = consolidations. ** The correlation is significant at the 0.01 level (two−tailed). * The correlation is significant at 0.05 level (two-tailed).

**Table 5:** Lung volumes quantified using the thoracic VCAR tool in terms of median, minimum, and maximum values obtained on baseline CT scans.

		EMP DX (%)	EMP SX (%)	EMP TOT (%/L)	HP DX (%)	HP X (%)	HP TOT (%/L)	GGO DX (%)	GGO SX (%)	GGO TOT (%/L)	OTHER DX (%)	OTHER SX (%)	OTHER TOT (%/L)	CONSOL DX (%)	CONSOL SX (%)	CONSOL TOT (%/L)	VOLUME DX (L)	VOLUME SX (L)	VOLUME TOT (L)
**All Patients**	**Median**	0.91	1.10	1.03	88.21	87.69	87.54	7.62	8.14	7.81	1.40	1.61	1.56	0.48	0.54	0.52	2.20	2.00	4.22
	**Minimum**	0.00	0.00	0.00	34.80	16.54	36.39	2.00	2.53	2.15	0.59	0.68	0.65	0.17	0.01	0.19	0.84	0.65	1.60
	**Maximum**	34.29	15.95	26.04	95.68	95.43	95.58	43.25	56.76	43.03	14.77	19.48	14.70	7.73	7.50	7.20	4.72	4.08	8.81
**RT-PCR positive**	**Median**	0.89	1.09	0.96	84.04	84.16	84.44	10.25	11.08	10.59	1.92	2.16	2.15	0.59	0.61	0.64	2.09	1.86	3.96
	**Minimum**	0.00	0.00	0.00	34.80	16.54	36.39	2.53	2.70	2.61	0.59	0.68	0.65	0.20	0.01	0.23	0.84	0.65	1.60
	**Maximum**	34.29	15.95	26.04	95.51	94.57	95.09	43.25	56.76	43.03	14.77	19.48	14.70	7.73	7.50	7.20	4.27	3.72	7.66
**Control group**	**Median**	0.91	1.11	1.08	92.38	91.62	92.01	4.52	4.59	4.62	1.03	1.11	1.07	0.34	0.42	0.39	2.32	2.19	4.65
	**Minimum**	0.00	0.04	0.05	75.40	69.80	73.75	2.00	2.53	2.15	0.60	0.70	0.65	0.17	0.21	0.19	1.25	1.05	2.30
	**Maximum**	4.06	5.57	4.76	95.68	95.43	95.58	21.87	27.25	23.58	2.45	2.94	2.60	0.77	0.93	0.84	4.72	4.08	8.81
***p* Value at Kuskal Wallis test**		0.278	0.270	0.229	**0.000**	**0.000**	**0.000**	**0.000**	**0.000**	**0.000**	**0.000**	**0.000**	**0.000**	**0.000**	**0.000**	**0.000**	**0.025**	**0.002**	**0.003**

Note. EMP = emphysema; HP = health parenchyma; GGO = ground-glass opacity; CONSOL = consolidations.

**Table 6:** Lung volumes quantified using the ANKE ASG-340 CT workstation pneumonia tool in terms of median, minimum, and maximum values obtained on baseline CT scans.

		EMP DX (%)	EMP SX (%)	EMP TOT (%/L)	HP DX (%)	HP SX (%)	HP TOT (%/L)	GGO DX (%)	GGO SX	GGO TOT (%/L)	OTHER DX (%)	OTHER SX (%)	OTHER TOT (%/L)	CONSOL DX (%)	CONSOL SX (%)	CONSOL TOT (%/L)	VOLUME DX (L)	VOLUME SX (L)	VOLUME TOT (L)
**All Patients**	**Median**	11.81	11.07	11.12	66.48	67.94	67.43	13.77	13.38	13.65	3.38	2.95	3.18	0.50	0.49	0.53	1.97	2.26	4.22
	**Minimum**	0.33	0.31	0.31	17.08	37.69	3.83	4.70	3.79	4.63	1.39	1.18	1.36	0.18	0.11	0.20	0.00	0.00	0.00
	**Maximum**	29.30	39.41	34.39	88.56	89.41	89.00	45.54	42.74	43.99	24.05	16.72	18.16	14.31	5.90	8.78	4.11	4.78	8.89
**RT-PCR positive**	**Median**	12.04	11.61	11.81	65.66	66.05	66.02	15.20	14.60	14.84	3.72	3.41	3.57	0.60	0.64	0.62	1.87	2.11	4.05
	**Minimum**	0.33	0.31	0.31	17.08	37.69	3.83	4.70	3.79	4.63	1.39	1.18	1.36	0.18	0.19	0.20	0.00	0.00	0.00
	**Maximum**	29.30	39.41	34.39	82.18	82.01	81.75	43.62	42.74	41.99	24.05	16.72	18.16	14.31	5.90	8.78	3.76	4.34	7.77
**Control group**	**Median**	11.27	9.75	10.77	69.46	71.52	70.79	9.78	10.24	10.02	2.49	2.40	2.44	0.38	0.34	0.35	2.13	2.37	4.61
	**Minimum**	1.29	1.08	1.24	43.62	45.18	43.35	5.04	5.89	5.80	1.56	1.53	1.56	0.23	0.11	0.22	1.06	1.29	2.35
	**Maximum**	25.29	24.04	24.54	88.56	89.41	89.00	45.54	42.67	43.99	8.15	8.03	8.08	1.39	1.32	1.34	4.11	4.78	8.89
***p* Value at Kuskal Wallis test**		0.102	0.058	0.083	0.315	0.199	0.220	**0.011**	**0.009**	**0.009**	**0.001**	**0.000**	**0.000**	**0.000**	**0.000**	**0.000**	0.579	0.720	0.777

Note. EMP = emphysema; HP = health parenchyma; GGO = ground-glass opacity; CONSOL = consolidations.

**Table 7:** Percentage change of quantified volumes by two tools between baseline and follow-up in patients with positive RT-PCR in terms of median, minimum, and maximum values.

		EMP DX (%)	EMP SX (%)	EMP TOT (%/L)	HP DX (%)	HP SX (%)	HP TOT (%/L)	GGO DX (%)	GGO SX (%)	GGO TOT (%/L)	OTHER DX (%)	OTHER SX (%)	OTHER TOT (%/L)	CONSOL DX (%)	CONSOL SX (%)	CONSOL TOT (%/L)	VOLUME DX (L)	VOLUME SX (L)	VOLUME TOT (L)
**ThoracicVCAR tool**	**Median**	13.25	19.46	12.90	−6.57	−6.25	−6.44	53.24	48.48	50.39	42.11	44.58	43.84	28.21	33.33	30.93	−19.91	−12.96	−16.99
	**Minimum**	−28.00	−7.56	−15.86	−13.70	−9.77	−14.88	−5.46	−7.53	−27.66	−31.67	−37.86	−38.82	−49.38	−31.08	−33.78	−30.47	−23.03	−27.59
	**Maximum**	100.00	100.00	100.00	39.07	98.73	44.02	95.03	92.19	92.05	94.37	94.95	94.66	94.16	94.10	93.48	67.67	73.08	65.45
**ANKE ASG-340 CT workstation pneumonia tool**	**Median**	−4.51	0.49	−1.02	−11.20	−10.69	−7.52	31.03	33.87	31.99	35.60	36.03	35.63	37.50	39.55	39.66	−11.13	−11.56	−11.17
	**Minimum**	−19.24	−20.39	−20.61	−13.95	−9.52	−18.0	−12.41	−11.05	−11.20	−7.78	−40.61	−56.60	−112.86	−12.37	−14.00	−15.77	−12.39	−16.71
	**Maximum**	97.86	98.91	98.39	26.78	18.84	36.30	96.79	87.18	86.09	91.14	87.46	89.21	97.55	93.59	96.13	34.31	32.64	33.47

Note. EMP = emphysema; HP = health parenchyma; GGO = ground-glass opacity; CONSOL = consolidations.

**Table 8:** Lung volumes quantified by two tools in terms of median, minimum, and maximum values obtained on follow-up CT scans.

		EMP DX (%)	EMP SX (%)	EMP TOT (%/L)	HP DX (%)	HP SX (%)	HP TOT (%/L)	GGO DX (%)	GGO SX (%)	GGO TOT (%/L)	OTHER DX (%)	OTHER SX (%)	OTHER TOT (%/L)	CONSOL DX (%)	CONSOL SX (%)	CONSOL TOT (%/L)	VOLUME DX (L)	VOLUME SX (L)	VOLUME TOT (L)
**ThoracicVCAR tool**	**Median**	0.86	1.14	1.06	92.27	91.65	92.01	4.31	4.68	4.52	0.98	1.05	1.00	0.39	0.41	0.39	2.61	2.32	4.89
	**Minimum**	0.00	0.00	0.00	54.70	1.04	50.39	0.67	2.66	2.47	0.60	0.67	0.64	0.21	0.22	0.22	0.88	0.70	1.21
	**Maximum**	28.59	14.05	22.11	96.27	95.78	96.04	37.82	47.13	42.35	5.66	5.24	5.46	1.80	1.78	1.79	4.22	9.96	7.73
**ANKE ASG-340 CT workstation pneumonia tool**	**Median**	12.85	12.73	12.85	72.77	73.63	70.95	8.26	8.30	8.20	2.13	2.01	2.09	0.35	0.39	0.37	2.31	2.61	4.83
	**Minimum**	0.25	0.13	0.19	45.85	52.44	41.75	0.33	4.08	4.13	0.64	1.20	1.25	0.18	0.18	0.18	0.00	0.00	0.00
	**Maximum**	28.62	36.91	33.20	91.08	91.74	91.43	43.36	36.36	39.74	8.20	8.69	8.45	39.00	1.69	1.68	3.79	4.29	7.80

Note. EMP = emphysema; HP = health parenchyma; GGO = ground-glass opacity; CONSOL = consolidations.

**Table 9:** ROC analysis results for volumes measurements obtained on baseline CT scans for both software.

	ThoracicVCAR							ANKE ASG-340 CT Workstation Pneumonia						
	AUC	Sensitivity	Specificity	Positive Predictive Value	Negative Predictive Value	Accuracy	Cut-off	AUC	Sensitivity	Specificity	Positive Predictive Value	Negative Predictive Value	Accuracy	Cut-off
EMP DX (%)	0.42	0.04	1.00	1.00	0.26	0.28	4.06	0.50	0.51	0.58	0.78	0.28	0.53	11.44
EMP SX (%)	0.44	0.07	0.98	0.89	0.26	0.29	4.31	0.51	0.70	0.43	0.79	0.32	0.63	7.61
EMP TOT %/L)	0.44	0.07	0.98	0.89	0.26	0.29	4.01	0.50	0.49	0.60	0.79	0.28	0.52	11.12
HP DX (%)	0.21	0.00	1.00	--	0.25	0.25	95.68	0.35	0.00	1.00	--	0.25	0.25	88.56
HP SX (%)	0.22	0.03	0.98	0.75	0.25	0.26	94.29	0.33	0.00	1.00	--	0.25	0.25	89.41
HP TOT (%/L)	0.21	0.00	1.00	--	0.25	0.25	95.58	0.34	0.00	1.00	--	0.25	0.25	89.00
GGO DX (%)	0.71	0.71	0.73	0.89	0.45	0.71	5.77	0.55	0.68	0.55	0.82	0.36	0.64	10.17
GGO SX (%)	0.70	0.70	0.73	0.88	0.45	0.71	5.74	0.55	0.68	0.53	0.81	0.35	0.64	10.26
**GGO TOT (%/L)**	**0.71**	**0.76**	**0.73**	**0.89**	**0.50**	**0.75**	**5.51**	0.55	0.68	0.55	0.82	0.37	0.65	10.03
OTHER DX (%)	0.71	0.48	0.98	0.98	0.38	0.60	1.89	0.60	0.50	0.75	0.86	0.33	0.56	3.64
OTHER SX (%)	0.71	0.52	0.90	0.94	0.38	0.61	1.92	0.61	0.25	0.98	0.97	0.30	0.43	6.42
OTHER TOT(%/L)	0.72	0.48	0.95	0.97	0.38	0.60	2.04	0.61	0.57	0.65	0.83	0.33	0.59	2.99
CONSOL DX (%)	0.72	0.55	0.88	0.93	0.39	0.63	0.50	0.64	0.64	0.63	0.84	0.37	0.64	0.42
**CONSOL SX (%)**	0.70	0.56	0.88	0.93	0.40	0.64	0.56	**0.68**	**0.77**	**0.55**	**0.84**	**0.44**	**0.71**	**0.35**
CONSOL TOT(%/L)	0.71	0.47	0.98	0.98	0.38	0.59	0.66	0.67	0.74	0.55	0.83	0.42	0.69	0.35
VOLUME DX (L)	0.38	0.04	0.98	0.83	0.25	0.28	3.65	0.37	0.00	1.00	--	0.25	0.25	4110.00
VOLUME SX (L)	0.34	0.00	1.00	NaN	0.25	0.25	4.08	0.39	0.00	1.00	--	0.25	0.25	4784.00
VOLUME TOT (L)	0.34	0.04	0.98	0.83	0.25	0.28	7.02	0.39	0.00	1.00	--	0.25	0.25	8894.00

Note. EMP = emphysema; HP = health parenchyma; GGO = ground-glass opacity; CONSOL = consolidations.

## Data Availability

All data are reported in the manuscript.
